# Assessment of the reliability of a non-invasive elbow valgus laxity measurement device

**DOI:** 10.1186/s40634-020-00290-2

**Published:** 2020-09-28

**Authors:** Kenneth Seiber, Chris Bales, Elisabeth Wörner, Thay Lee, Marc R. Safran

**Affiliations:** 1 Department of Orthopaedic Surgery, Stanford University, 430 Broadway, MC 6342, Redwood City, CA 94063 USA; 2 Orthopaedic Biomechanics Laboratory, Congress Medical Foundation, Pasadena, CA USA

**Keywords:** Elbow tester, Ulnar collateral ligament, Pitchers elbow, Valgus stress insufficiency, Valgus laxity, Elbow, Valgus stress

## Abstract

**Purpose:**

The purpose of this study was to assess the reliability of a new objective measurement tool to measure the valgus stress laxity of the ulnar collateral ligament (UCL) of the elbow, the “Elbow Tester”. The anterior oblique portion of the ulnar collateral ligament (UCL) of the elbow is the primary static restraint to valgus stress during the overhead throwing motion. The main research question was if the “Elbow Tester” that we have developed was reliable and reproducible for further use in research and daily practice.

**Methods:**

Three different examiners tested both elbows of 11 volunteers for UCL laxity. Each elbow was tested 5 times using a standard 2 Nm valgus load, and 3 times using a manual maximum valgus load. One examiner tested the volunteers again 1 week later. The outcomes of elbow valgus laxity were compared between examiners. The intraobserver reliability was assessed using an intraclass correlation coefficient (ICC) and interobserver reliability was also assessed with a mixed model repeated ANOVA test.

**Results:**

The device demonstrated a high level of intraobserver reliability with both the 2 Nm valgus force and manual maximum valgus force, using a minimum of three trials as determined by an ICC > 0.9 for all examiners. The interobserver reliability was moderate using the 2 Nm valgus load with an ICC value of 0.72 and significant different outcomes of elbow valgus laxity amongst examiners (*p* < 0.01). A high interobserver reliability (ICC value of 0.90) was observed using manual maximum valgus force and no differences between outcomes (*p* > 0.53).

**Conclusion:**

The noninvasive valgus elbow tester demonstrates high interobserver and intraobserver reliability using manual maximum valgus force and can be used for further research and daily practice.

## Background

The anterior bundle of the ulnar collateral ligament (UCL) is commonly referenced to as the primary restraint to valgus stress of the elbow between 30° and 120° of elbow flexion [1]. The UCL is subject to repetitive, near-failure stresses during the overhead throwing motion, which can result in acute injury or progressive microtrauma [2, 3]. This has been noted to occur in athletes that participate in overhead throwing sports such as baseball, volleyball, tennis, javelin, water polo and football [4]. Disruption of the UCL can cause significant pain and can result in the inability to throw or play. UCL insufficiency may result in frank instability symptoms of the elbow and injury to other structures in and about the elbow, such as the ulnar nerve, flexor-pronator muscle tendon group, or posteromedial or lateral elbow joint surfaces. Increased valgus elbow laxity, due to UCL insufficiency, has been recognized as a significant adverse result of extensive pitching in baseball at professional and even recreational levels of competition [5].

The diagnosis of injury to the ulnar collateral ligament is important but can be challenging in daily practice. The subtle changes that can be detected clinically can lead to diagnoses that are underappreciated or inaccurate. Suspicion for a UCL tear is based on symptoms of pain while throwing, notably during the late cocking and acceleration phases of throwing. Some pitchers do not experience pain, they rather experience loss of effectiveness as a result of diminished accuracy of their throw or declining throwing velocity [4]. Detecting actual UCL insufficiency from sports-related increased valgus laxity is challenging, even for an experienced specialist. Provocative tests such as the manual valgus stress test, moving valgus stress test, and milking maneuver help recreate symptoms, but also can be non-specific, and influenced by other diagnoses such as flexor-pronator strains, intra-articular lesions, medial epicondylar apophysitis, and stress fractures [2]. Additional imaging is often necessary. MRI with intra-articular contrast, MRA, has to date the highest sensitivity and specificity to detect UCL tears [6]. An MRI without contrast is generally considered to have low sensitivity [7, 8]. Additionally, the integrity or laxity of the ulnar collateral ligament is not assessed as it is a static image of the elbow. There is an increasing interest in using ultrasound or radiography for objective imaging of UCL insufficiency [7, 9]. UCL insufficiency can be diagnosed by ultrasound in combination with a dynamic stress test. Sasaki et al. described the use of stress sonography to assess medial elbow joint laxity in baseball players, where significant medial elbow joint gapping and structural changes to the ligament were detected [10]. Other studies also showed that ultrasound is a reliable and precise method for detecting changes in ulnohumeral joint gapping and the UCL ligament [11, 12]. Musculoskeletal sonography is however an operator and position dependent imaging technique for assessing UCL insufficiency [13]. Radiography in combination with a Telos elbow stress tester device can also be used to assess the medial joint gap while applying valgus force [14–16]. Conway and Thompson both demonstrated a high specificity in positive stress radiographs with asymmetric joint line opening of 2 or more mm [7, 17]. Disadvantages of this imaging technique are that it requires bilateral elbow radiographs to detect a subtle asymmetric joint opening and that it is subject to a significant interobserver and intraobserver measurement error [18].

There is yet to be a reliable, reproducible, and objective measurement device used in current practice to detect UCL insufficiency, that is noninvasive and non-irradiating. A noninvasive elbow valgus laxity measurement device was developed by the senior author, from here on referred to as the “*Elbow Tester*”. The following study was designed to test the interobserver and intraobserver reliability of this unique Elbow Tester.

## Materials and methods

This study was approved by the University Institutional Review Board.

### Elbow tester and examiners

**Fig. 1:**
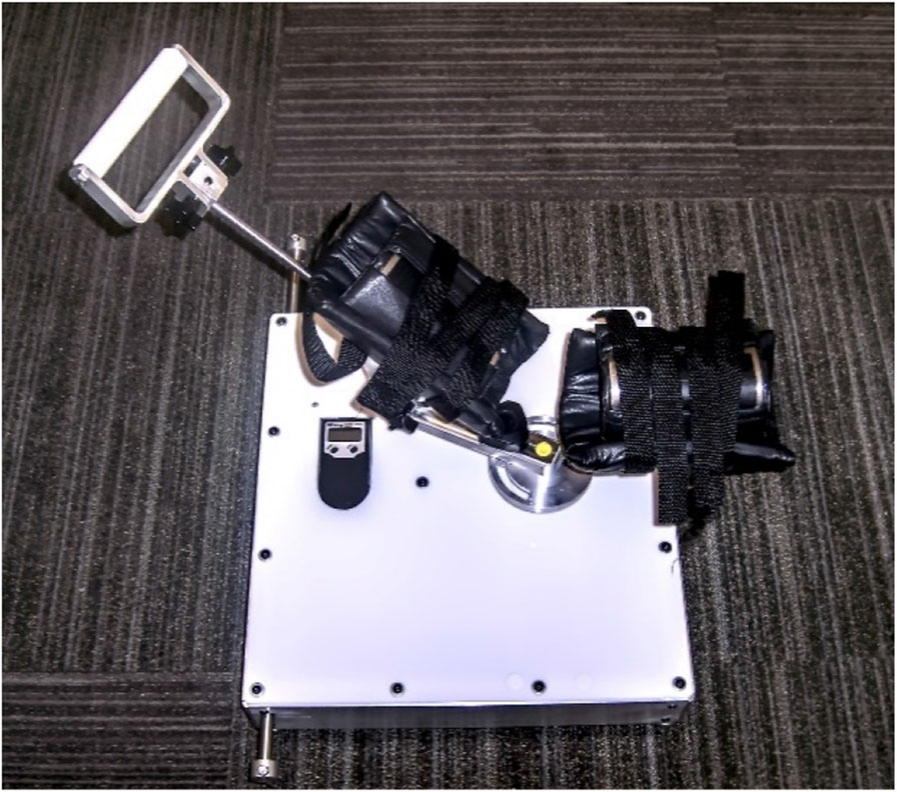
The Elbow Tester

**Fig. 2:**
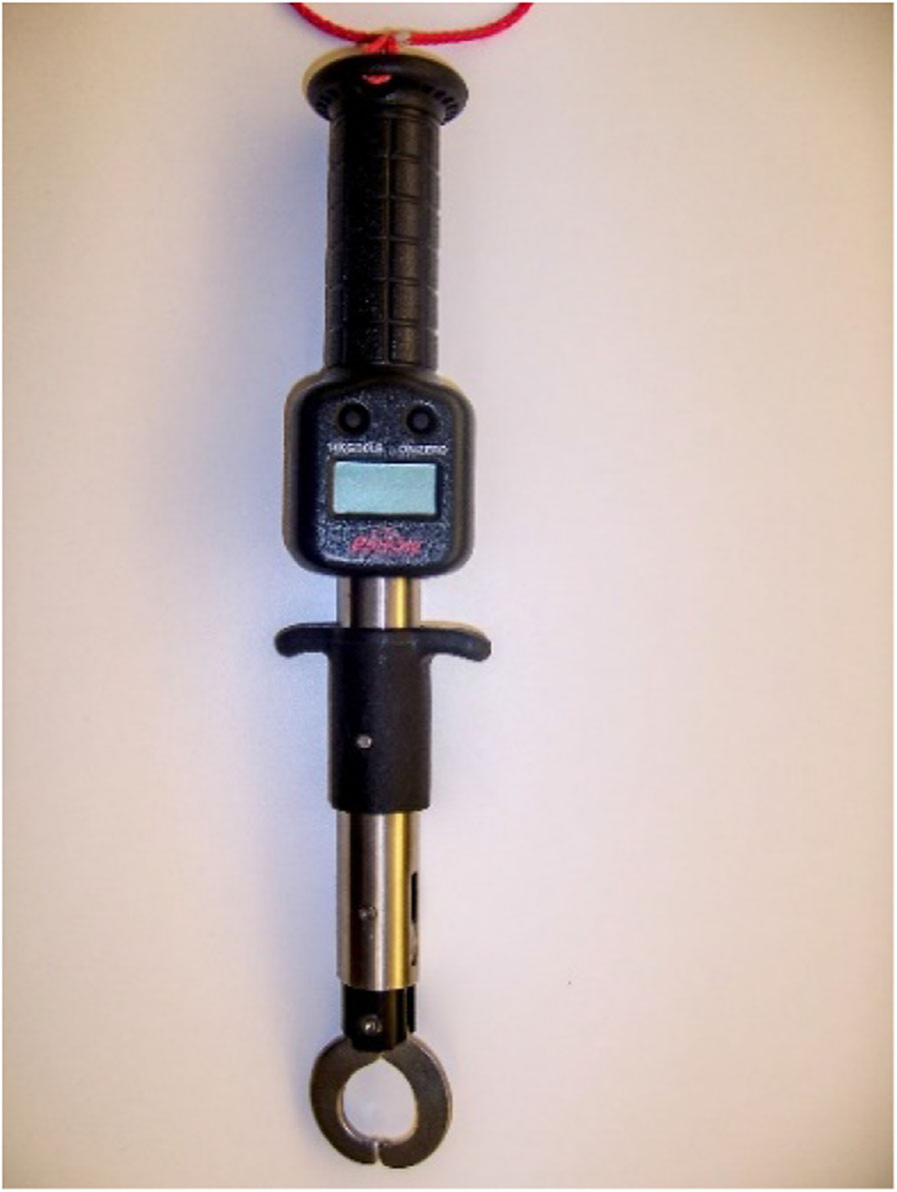
Digital force measurement device

**Fig. 3:**
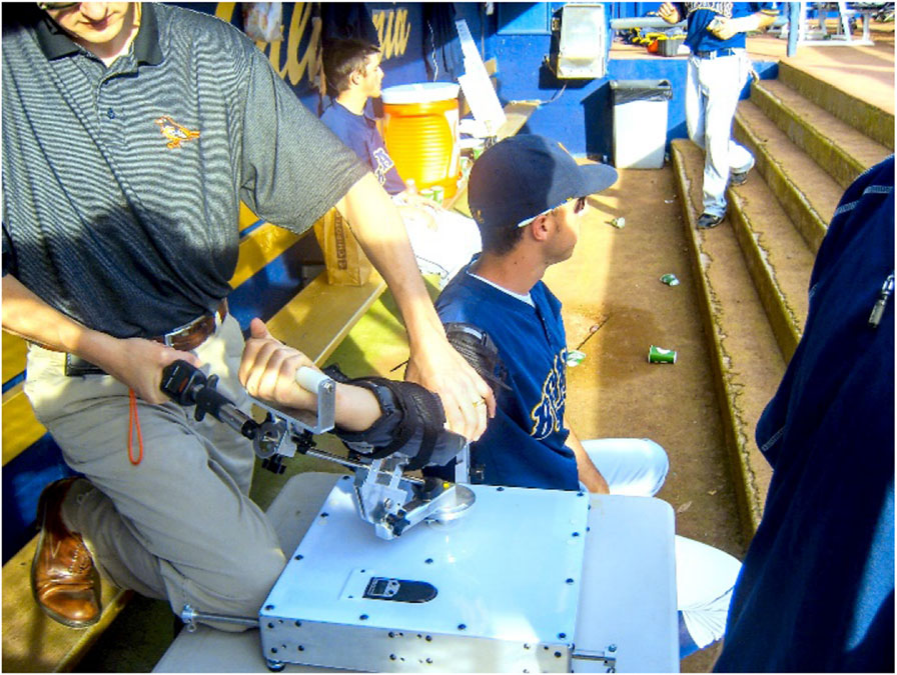
Testing volunteer with the Elbow Tester

The Elbow Tester is an apparatus developed by the senior author in conjunction with a biomechanical engineer. The testing device is portable and can be placed on a countertop. The subject’s arm is positioned into the padded arm and forearm clamps (Fig. 1). In this study various valgus loads were applied across the elbow with a digital force measurement device (Fig. 2) and the angular displacement of the forearm was measured using a three-dimensional coordinate measurement device (MicroScribe™ G2, Immersion Corp., San Jose, CA, USA). The device caused minimal discomfort to the subject. The loads applied were first a 2 Nm force to a provide a consistent and accurate applied torque. This was followed by a manual maximum force which was stopped at the sensation of tightness of the elbow of the subject. The applied loads were far below the physiologic loads created by the subject during actual throwing and below documented failure limits for isolated cadaveric ligament specimens [1, 19, 20]. The subject remained completely alert and oriented during the assessment with full visual and tactile appreciation of the stresses applied to their arm, as occurs during normal physical examination of the elbow (Fig. 3).

Three examiners (KS, CB, MS) participated in the study to test the Elbow Tester device. The principle investigator (MS) has extensive clinical experience with UCL deficient patients and with surgical reconstructions. The other two examiners (KS, CB) were fellows in orthopedic sports medicine with proficiency in musculoskeletal examination.

### Participants

Healthy volunteers were included in the study after signing informed consent. They completed a simple questionnaire and both their elbows underwent a physical exam. Exclusion criteria were elbow arthritis, elbow surgery, relevant systemic musculoskeletal disorder, or significant past or current injury of the elbow other than possible injury to the UCL. There was one baseball player with an UCL injury that was included as a pathologic elbow. A total of 11 subjects were enrolled, averaging 33.5 years of age. Eight were male, three were female, and 9 were right-handed and 2 were left-handed. Both elbows of each subject were examined by a board-certified orthopedic surgeon for range of motion and stability. Stability was assessed using the classic Jobe valgus stress test, the modified Milking Maneuver, and the Moving Valgus Stress Test [21–24]. The elbows of all 9 volunteers were pooled because this study was conducted for reproducibility and consistency between multiple tests and testers, not to compare results between patients or elbows.

### Testing protocol

The procedure for each instrumented measurement with the “Elbow Tester” was as follows:

1. The subject was seated with their shoulder abducted approximately 70 degrees in neutral rotation. The arm was placed and secured in the measurement device with the elbow centered over the point of rotation, 30 degrees flexion, with the shoulder in neutral rotation. This degree of elbow flexion within this range has been shown to be independent in assessment of valgus stability in cadaveric studies [25]. Likewise, neutral rotation of the forearm has also been shown to allow for maximal potential valgus laxity [1].
2. The arm rested in its neutral position and a position measurement was taken with the elbow in the neutral position using a three-dimensional coordinate measurement device (MicroScribe™ G2, Immersion Corp., San Jose, CA, USA).
3. In order to provide a consistent and accurate applied torque, 1.5 pounds of valgus force (2 Nm) was applied using a digital Berkley Fish Scale (Berkley, Spirit Lake, Iowa) (Fig. 2) while the valgus laxity was measured in degrees using the MicroScribe™ G2 measurement device. The angular displacement of the forearm was then calculated from the neutral position to the valgus loaded position.
4. Each elbow was tested 5 times in this manner.
5. The elbows were then tested three times as above but using a manual maximum valgus torque across the elbow. The maximum torque was measured when the force was stopped at a sensation of “tightness” of the subject, as would be done during typical physical examination. The manual maximum - until tightness - is commonly used with the KT-1000 arthrometer for the knee to assess anterior tibial displacement as a determinant of ACL integrity or tear and was found to be the most accurate and reproducible for side to side difference measurements [26].

Each subject was tested by two examiners (MS, KS) and 6 were tested by a third examiners (CB) as CB was only partly available to test participants. One examiner (KS) tested each of the 11 subjects twice, 1 week apart, to assess the intraobserver reliability further over separate testing attempts.

The valgus laxity of the right and left elbow for each patient were calculated for all the trials. The intraclass correlation coefficient (ICC) was used to measure the intraobserver reliability. This evaluates the proportion of variance of an observation due to between-subject variability in the true scores [27]. A ICC value greater than or equal to 0.75 indicates a good reliability. Values between 0.5 and 0.75 indicate moderate reliability [28]. The ICC value was determined for 5 trials of a 2 Nm valgus load, 3 trials of a 2 Nm valgus load (first 3 trials of the 5), and 3 trials using the manual maximum valgus load. The overall interobserver reliability was determined using a mixed model repeated ANOVA.

## Results

### Intraobserver reliability

The ICC value for each examiner was determined for the 5 trials of a 2 Nm valgus load. The ICC values for all three examiners (the intraobserver reliability) were higher than 0.95, which indicates a high level of reliability across the 2 Nm trials using 5 attempts.

The ICC value for each examiner was likewise determined using just the first 3 trials of the 2 Nm valgus load to see if a high degree of intraobserver reliability still was present with less loading attempts. The ICC values for each examiner over three trials were all greater than 0.93, indicating that a reliable reading can be made through a minimum of three trials.

Finally, the ICC value for each examiner using 3 trials with the manual maximum force applied to the elbow was determined. The values ranged from 0.93 to 0.98, which also indicates a high level of reliable results for all three examiners over three maximum force trials.

One rater tested the elbows twice. The ICC value of the test – retest procedure was 0.89 (95% CI, 0.75–0.95) (Tables 1, 2, and 3).

**Table 1:** Intraclass correlation coefficient of 5 trials with 2 Nm valgus load (elbows pooled)

ICC 5 trials with 2 Nm valgus load	
Examiner	ICC
KS (1st attempt)	ICC = 0.954 (95% CI, 0.898–0.973)
KS (2nd attempt)	ICC = 0.964 (95% CI, 0.919–0.979)
CB	ICC = 0.952 (95% CI, 0.872–0.980)
MS	ICC = 0.945 (95% CI, 0.868–0.972)

**Table 2:** Intraclass correlation coefficient of first 3 out of 5 trials with 2 Nm valgus load

ICC first 3 trials (out of 5) with 2 Nm valgus load	
Examiner	ICC
KS (1st attempt)	ICC = 0.933 (95% CI, 0.815–0.955)
KS (2nd attempt)	ICC = 0.955 (95% CI, 0.873–0.970)
CB	ICC = 0.958 (95% CI, 0.848–0.980)
MS	ICC = 0.980 (95% CI, 0.863–0.976)

**Table 3:** Intraclass correlation coefficient of 3 trials with manual maximum load

ICC 3 trials with manual maximum load	
Examiner	ICC
KS (1st attempt)	ICC = 0.975 (95% CI, 0.916–0.985)
KS (2nd attempt)	ICC = 0.970 (95% CI, 0.900–0.982)
CB	ICC = 0.934 (95% CI, 0.769–0.968)
MS	ICC = 0.983 (95% CI, 0.902–0.983)

### Interobserver reliability

The reliability between examiners had an ICC value of 0.72 using a 2 Nm valgus force (95% CI, 0.64–0.95), a moderate interobserver reliability. The examiners obtained statistically different valgus angular displacement values using the 2 Nm valgus load (*p* < 0.01). On the contrary, using a manual maximum valgus force a high interobserver reliability ICC value of 0.90 (95% CI, 0.60–0.98) was found. This indicated a high similarity of the valgus laxity outcomes between examiners performing a manual maximum trial. There were no statistical differences between measurements using the manual maximum valgus force (*p* > 0.53).

The outcomes are in normal range for reproducibility. This is a novel way to measure UCL laxity, thus there are currently no data available in the literature measuring degrees of displacement of the forearm for comparison.

## Discussion

This study was conducted to assess the reproducibility of a newly developed Elbow Tester. The main finding of this study is that the newly developed Elbow Tester is able to produce high interobserver and intraobserver reliability of the measurement of elbow valgus laxity when using a manual maximum valgus force. This is consistent with current literature on the KT-1000 knee ligament arthrometer to assess anterior or posterior cruciate ligament insufficiency, where manual maximum force was found to be the most reliable [26].

The volunteers tested were medical students, nurses, ancillary health care professionals with no history of injury or surgery to their elbows. Additionally, there was one baseball player with an UCL injury that was included as a pathologic elbow. Due to the fact that the elbows were not compared between subjects or within subjects, it was thought that the pathologic elbow would not affect the outcomes of the study and was therefore included in the study. An a priori power analysis was not performed, as there is no prior data that assessed valgus laxity of the elbow in this manner. Based on the data, a post hoc analysis suggests this was not underpowered (B = 0.87, alpha level = 0.05).

The current available imaging techniques such as stress sonography and radiography of the elbow can measure significant differences between pathologic and normal elbows, but fail to show high reliability outcomes [16, 18]. Magnetic resonance arthrogram (MRA) is the imaging technique of choice to identify injury to the UCL ligament, but it is a static test and not a dynamic assessment of the ligament [29]. It is also an invasive and expensive imaging technique that is in most clinics not immediately available. Thus, there was a need for a more reliable, reproducible, objective measurement tool for the assessment of UCL insufficiency of the elbow. The Elbow Tester showed a high reproducibility and similarity of outcome measures for assessing valgus laxity of the elbow.

The high reproducibility of this study suggests that it can be used amongst clinicians with little experience in diagnosis of elbow injuries. The device is portable and can be easily used in daily practice in the outpatient clinic. The Elbow Tester can be of use to test UCL insufficiency of the elbow in the post-reduction management after dislocation as this is often based on subjective clinical examination. It can also be of use in throwing athletes who experience elbow pain or experience a loss of velocity and efficiency while throwing. The Elbow Tester can potentially identify athletes at risk for UCL injuries. It may allow clinicians to better understand injuries to the ulnar collateral ligament – either due to trauma or microtrauma – and choose the right treatment.

Future research is suggested to assess the validity of the Elbow Tester by comparison to other imaging techniques for diagnosing UCL insufficiency. It is also suggested to compare pathologic elbows and normal elbows with the Elbow Tester.

## Conclusion

Our study suggests that the newly developed elbow valgus stress tester is a reliable measurement tool for the assessment of UCL laxity when using a manual maximum valgus force. It is a reproducible objective measurement device that can be used in future research and daily practice in patients with suspected UCL insufficiency of the elbow.

No funding was received for this study. There were no competing interests. All authors made substantial contributions to the design, testing or analysis and interpretation of the data. Each author approved the submitted version and have agreed both to be personally accountable for the author’s own contributions and to ensure that questions related to the accuracy or integrity of any part of the work, even ones in which the author was not personally involved, are appropriately investigated, resolved, and the resolution documented in the literature.

## Abbreviations

UCL: Ulnar collateral ligament
ICC: Intraclass correlation coefficient
MRI: Magnetic Resonance Imaging
ANOVA: Analysis of Variance
